# Shear Wave Elastography in Patients with Primary and Secondary Hyperparathyroidism

**DOI:** 10.3390/jcm10040697

**Published:** 2021-02-10

**Authors:** Daniela Amzar, Laura Cotoi, Ioan Sporea, Bogdan Timar, Oana Schiller, Adalbert Schiller, Andreea Borlea, Nicusor Gheorghe Pop, Dana Stoian

**Affiliations:** 1 Endocrinology Department, “Victor Babes” University of Medicine and Pharmacy Timisoara, 2nd Eftimie Murgu Square, 300041 Timisoara, Romania; amzar.danielageorgiana@gmail.com (D.A.); stoian.dana@umft.ro (D.S.); 2 PhD School Department, “Victor Babes” University of Medicine and Pharmacy Timisoara, 2nd Eftimie Murgu Square, 300041 Timisoara, Romania; borlea.andreea@umft.ro; 3 Internal Medicine 2nd Department, “Victor Babes” University of Medicine and Pharmacy Timisoara, 2nd Eftimie Murgu Square, 300041 Timisoara, Romania; isporea@umft.ro (I.S.); schiller.adalbert@gmail.com (A.S.); 4 Internal Medicine 3th Department, “Victor Babes” University of Medicine and Pharmacy Timisoara, 2nd Eftimie Murgu Square, 300041 Timisoara, Romania; bogdan.timar@umft.ro; 5 Dialysis Medical Center B Braun Avitum, 636 Remetea Mare, 307350 Timisoara, Romania; oana.schiller@bbraun.com; 6 Centre for Modelling Biological Systems and Data Analysis, Department of Functional Sciences, “Victor Babes” University of Medicine and Pharmacy Timisoara, 2nd Eftimie Murgu Square, 300041 Timisoara, Romania; pop.nicusor@umft.ro

**Keywords:** elastography, primary hyperparathyroidism, secondary hyperparathyroidism, thyroid ultrasound, shear wave elastography

## Abstract

Objectives: In this study, we aim to determine the elastographic characteristics of both primary and secondary hyperparathyroidism using shear wave elastography. We also aim to evaluate the elastographic differences between them, as well as the differences between the parathyroid, thyroid, and muscle tissue, in order to better identify a cutoff value for the parathyroid tissue. Methods: In this prospective study, we examined a total of 68 patients with hyperparathyroidism, divided into two groups; one group consisted of 27 patients with primary hyperparathyroidism and the other group consisted of 41 selected patients with confirmed secondary hyperparathyroidism. The elasticity index (EI) was determined in the parathyroid, thyroid, and muscle tissue. The determined values were compared to better identify the parathyroid tissue. Results: The median value of mean SWE values measured for parathyroid adenomas from primary hyperparathyroidism was 4.86 kPa. For secondary hyperparathyroidism, the median value of mean SWE was 6.96 KPa. The median (range) presurgical values for parathormone (PTH) and calcium were 762.80 pg/mL (190, 1243) and 9.40 mg/dL (8.825, 10.20), respectively. We identified significant elastographic differences between the two groups (*p* < 0.001), which remained significant after adjusting elastographic measures to the nonparametric parameters, such as the parathormone value and vitamin D (*p* < 0.001). The cutoff values found for parathyroid adenoma were 5.96 kPa and for parathyroid tissue 9.58 kPa. Conclusions: Shear wave elastography is a helpful tool for identifying the parathyroid tissue, in both cases of primary and secondary hyperparathyroidism, as there are significant differences between the parathyroid, thyroid, and muscle tissue. We found a global cutoff value for the parathyroid tissue of 9.58 kPa, but we must keep in mind that there are significant elastographic differences between cutoffs for primary and secondary hyperparathyroidism.

## 1. Introduction

Recent advancements in medical technologies have improved diagnostic methods, thus increasing the incidence of certain endocrine diseases [1,2]. Hyperparathyroidism is a common endocrine disorder, as primary hyperparathyroidism is the third endocrinopathy after type 2 diabetes mellitus and thyroid disease [3], parathyroid adenoma being cited as the most common cause of primary hyperparathyroidism. Other rare causes such as multiglandular disease and parathyroid carcinoma are cited [4,5,6]. Secondary hyperparathyroidism, as a complication of chronic kidney disease, presents a high prevalence among patients on hemodialysis [7,8].

Primary hyperparathyroidism is mostly found in asymptomatic forms as high serum parathormone (PTH) concentrations and consequent high serum calcium concentrations, diagnosed by active screening [1,4,5,6,9,10,11]. The high prevalence is seen among postmenopausal women (female–male ratio 3–4:1) [1,12,13,14], emphasizing the fact that incidence increased because of active screening, as shown by a study conducted by Palmer et al. in Sweden [15].

The disturbances of calcium, phosphate, and vitamin D present in patients with chronic kidney disease determine high concentrations of serum parathormone (PTH) and consecutively lead to secondary and potentially tertiary hyperparathyroidism [16,17,18]. Prevalence studies have shown high numbers among patients with chronic kidney disease and renal replacement therapy—43.8% in France, 46.8% in Russia, and 42.9% in the United Kingdom. The United States has a prevalence of 54% [7].

Regardless of the etiology of hyperparathyroidism, primary or secondary, surgery represents a valid, confirmed, and corrective treatment in both cases. Minimally invasive parathyroidectomy (MIP) is cited as a preferred approach in many specialized centers [12,16,17,18,19]. Hence, the need to correctly identify the number and location of affected glands is present, making parathyroid ultrasonography a mandatory stage in the identification and treatment for both primary and secondary hyperparathyroidism.

Given the positive aspects of ultrasonography, such as its noninvasive character, reproducibility, ease of manipulation, high resolution in real-time, harmlessness to children and pregnant women, and lack of X-ray exposure or need of contrast agents, it is the most accessible and cost-efficient imaging technique for identifying thyroid disease [20,21]. Complementary to ultrasonography, elastography has been a turning point in the field of medical imaging. Labeled as “palpation imaging”, elastography offers qualitative and quantitative information on the tissue anatomical architecture, identifying changes in tissue stiffness [20,22,23,24]. Elastography has been validated as a marker of pathological states in many clinical fields by facilitating the diagnosis, differential diagnosis, and therapeutic management and establishing its role in endocrinology (thyroid and parathyroid area) [25,26], hepatogastroenterology [20,27,28,29], senology [30,31], urology [32,33,34], and otorhinolaryngology [35].

Shear wave elastography generates shear waves in the targeted tissues using the acoustic radiation force and tracks the propagating shear waves using ultrasound imaging techniques. The speed of the wave is then spatially mapped. The speed of the shear wave is directly related to the local stiffness of the tissue. It allows real-time monitoring of shear waves in 2D by measuring shear wave speed or Young’s modulus *E* and generating quantitative elastograms [36].

In shear wave elastography, shear waves have a perpendicular motion to the direction of wave propagation, defined by shear modulus *G* and directly measured by shear wave speed (*cS*), which can be reported in m/s or converted to Young’s modulus *E* in kilopascals (kPa) [2,22,36].

Used in both primary and secondary hyperparathyroidism, elastography proved in our previous studies [25,37] to be an accurate predictor of parathyroid tissue when compared to thyroid or muscle tissue. For differential diagnosis in cases of coexistence of benign thyroid lesions, it still is an issue to be discussed in further studies.

The objective of this prospective study was to determine the characteristics of parathyroid adenomas in primary hyperparathyroidism and the characteristics of hyperplastic parathyroid glands in patients with chronic kidney on renal replacement therapy, using shear wave elastography, to determine the elastographic differences, and also to identify a cutoff value for the parathyroid tissue, adding value to the presurgical identification and differential diagnosis.

## 2. Material and Methods

In this prospective study, we evaluated a total of 68 patients, divided into two groups of patients diagnosed with primary and secondary hyperparathyroidism.

The first group, evaluated from October 2018 to December 2019, consisted of 27 patients diagnosed with primary hyperparathyroidism. Primary hyperparathyroidism was identified through biochemical evaluation, Technetium Sestamibi scintigraphy (MIBI), and 2B-ultrasound examination and confirmed by a pathology report, considered the gold standard for diagnosis. All patients were adults and written consent was given. We excluded ectopic parathyroid adenomas diagnosed by MIBI scintigraphy.

The second group consisted 41 patients from the B Braun Dialysis Center from May 2019 to June 2019. All patients presented with end-stage renal disease and were on chronic hemodialysis treatment, clinically stable, and without any acute intercurrence. Hemodialysis was performed three times a week, the mean duration of hemodialysis therapy being 5.6 +/− 4.89 years.

We selected 41 patients out of the one hundred and twenty-three evaluated. From the one hundred and twenty-three evaluated, fifty-nine patients had secondary hyperparathyroidism confirmed by clinical, biological evaluation, and MIBI scintigraphy or MRI for more than six months. 

From 59 patients identified with secondary hyperparathyroidism, 25 presented with multiple hyperplastic parathyroid glands (2–4 parathyroid hyperplasia). From these 25 patients, we selected only the gland with the biggest diameter, that is, the gland with the highest elasticity index, for the final analysis. Patients with total thyroidectomy were excluded from the study.

The study was approved by the Ethics Committee, of the Dialysis Center (IIT2/24 April 2019), and all patients signed a written informed consent. The study followed the Ethics Code of the World Medical Association (Declaration of Helsinki, Seoul, Korea, October 2008).

### 2.1. Conventional Ultrasound

A 2B-mode ultrasound evaluation of the parathyroid glands was performed in all patients with an Aixplorer Mach 30 machine (SuperSonic Imagine, Aix-en-Provence, France) using a linear, high-resolution transducer of 15–4 or 18–5 MHz chosen depending on the clarity of the image; profound parathyroid glands were evaluated with a 15–4 probe, thus obtaining better images in these cases. The patient was examined in a supine position with hyperextension of the neck, maintaining regular breathing. The following parathyroid parameters were evaluated: localization, form, parathyroid dimensions, and volume. The vascular rim was examined using Color Doppler ultrasound. Parathyroid ultrasound evaluation was performed by two practitioners individually, with one of them having extensive experience in neck ultrasound and elastography.

### 2.2. Elastography Examination

After performing conventional ultrasound, the examiner performed two-dimensional shear wave elastography (2D-SWE) with an Aixplorer system (SuperSonic Imagine, France) using the same linear, high-resolution transducers, with the patient maintaining the supine position and neck hyperextension. 

**Figure 1:**
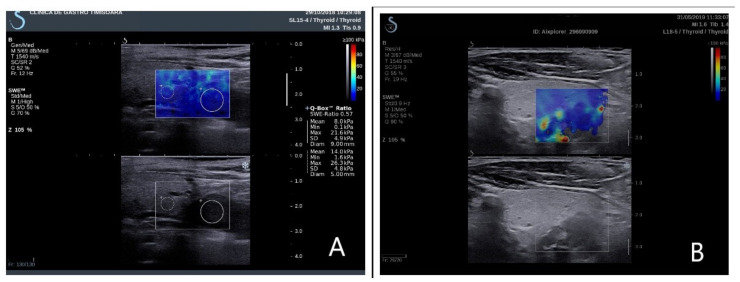
Elastograms overlapping on the B-mode image. (**A**) Right inferior parathyroid adenoma in primary hyperparathyroidism; (**B**) right inferior parathyroid hyperplasia in secondary hyperparathyroidism.

The examiner maintained precise adherence for a minimal 6 s to the probe on the examination area, not applying any manual compression, permitting the transducer to induce acoustic vibrations in the parathyroid tissue; after image stabilization, a real-time elastogram overlapped on the B-mode image, determining a color map (Figure 1). After image stabilization, on a frozen image, quantitative measurements were performed on parathyroid tissue, thyroid, and muscle tissue. All patients were examined by two clinicians, one with over 15 years of experience in elastography and neck ultrasound. Five measurements were performed and recorded for each parathyroid gland evaluated. We determined and analyzed the median results of all the quantitative measurements. 

**Figure 2:**
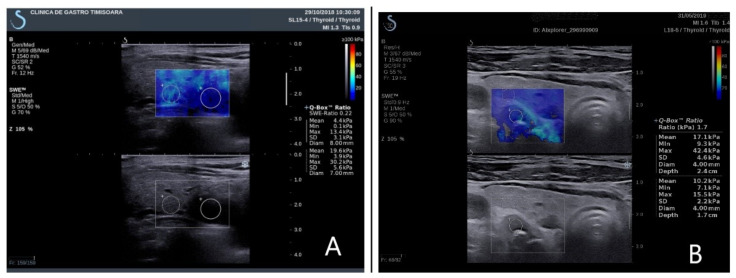
Elasticity index parathyroid and thyroid tissue. (**A**) Right inferior parathyroid adenoma in primary hyperparathyroidism; (**B**) left inferior parathyroid hyperplasia in secondary hyperparathyroidism.

Quantitative information, described as the elasticity index (EI), was obtained on the frozen elastogram image using a quantification box (Q-box), placed in the regions of interest (ROI) (Figure 2). The elasticity parameters are displayed after software computing evaluating the mean SWE, minimum SWE, maximum SWE, and standard deviation of elasticity value. All measurements are numerically expressed in kilopascals (kPa). As there are no scale settings for the parathyroid examination, a thyroid scale was used (0–100 kPa).

### 2.3. Statistical Analysis

We performed descriptive and inferential statistical analysis to summarize the characteristics of the study population. The results of the Shapiro–Wilk normality test showed a non-Gaussian distribution. Continuous variables were presented as median and interquartile range (Q1–Q3) and categorical variables were presented as frequency and percentages. To compare patients’ characteristics, we used the Mann–Whitney *U* test for numeric variables and the Fisher exact test for nominal variables. For comparing the SWE values of parathyroid, thyroid, and muscle tissue, we used the Kruskal–Wallis test. A characteristic (ROC) curve was employed to illustrate the identification ability, and the thresholds to discriminate between primary/secondary hyperparathyroidism and the parathyroid and thyroid muscles were determined with the Youden’s index. A *p*-value of < 0.05 was considered to indicate a statistically significant difference. Data analysis was performed using SPSS 26 (Statistical Package for the Social Sciences, Chicago, IL, USA).The study was approved by the Ethics Committee of our hospital and all patients signed a written consent. 

## 3. Results

We evaluated a total of 68 patients, divided into two lots, one of 27 patients with primary hyperparathyroidism and the other lot consisting of 41 patients with secondary renal hyperparathyroidism.

The baseline characteristics (median values) of the two groups studied are presented in Table 1 and Table 2.

A total number of 68 parathyroid glands were evaluated, 27 parathyroid adenomas and 41 parathyroid hyperplasia from secondary renal hyperparathyroidism. Five measurements were performed for each parathyroid gland and compared to the surrounding healthy thyroid tissue and muscle tissue. In Table 3, we present our elastography results (median values) for the first lot of primary hyperparathyroidism; in Table 4, we present the result for the second lot of secondary renal hyperparathyroidism. We took into consideration the mean SWE value, minimum, and maximum SWE value for parathyroid, thyroid, and muscle tissue in each lot.

Upon comparing the results of the two studied lots, we found that there were statistically significant differences of sex distribution between the two studied lots (*p* < 0.001, Fischer exact test).

The pathophysiology pathways are different between primary and secondary hyperparathyroidism. The first, the pathophysiological mechanism, arises from a somatic mutation, enhancing parathyroid cellular proliferation and changing the set point of the calcium-sensing receptor; the mechanism is still unclear at the moment [38]. In the second, changes are induced by continuous stimulation of the parathyroid gland, combining elevated serum phosphorus and decreased extracellular calcium concentration as well as low levels of calcitriol, inducing PTH synthesis and release. Elevated levels of FGF-23 also exacerbate the deficiency in vitamin D, acting as an additional factor to secondary hyperparathyroidism. Even in the early stages, there is a decrease of the calcium sensor receptor and vitamin D receptor, making parathyroid cells unresponsive to ambient calcium and vitamin D levels, resulting in a proliferative state of parathyroid cells and inducing parathyroid hyperplasia [19,39].

Keeping these pathophysiology changes in mind, we conducted diagnostic tests to evaluate the elastographic differences between the two types of hyperparathyroidism. The results are presented in Table 5.

**Figure 3:**
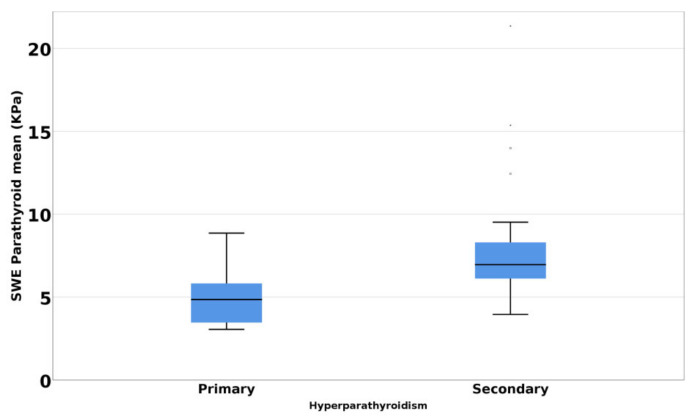
Mean SWE elastography values in primary hyperparathyroidism versus secondary hyperparathyroidism.

The Mann–Whitney *U* test demonstrated a statistically significant difference (*p* < 0.001) of SWE mean parathyroid tissue between primary and secondary hyperparathyroidism. Higher values were observed in the secondary hyperparathyroidism group (Figure 3). The difference remains significant even after PTH values were adjusted for age.

**Figure 4:**
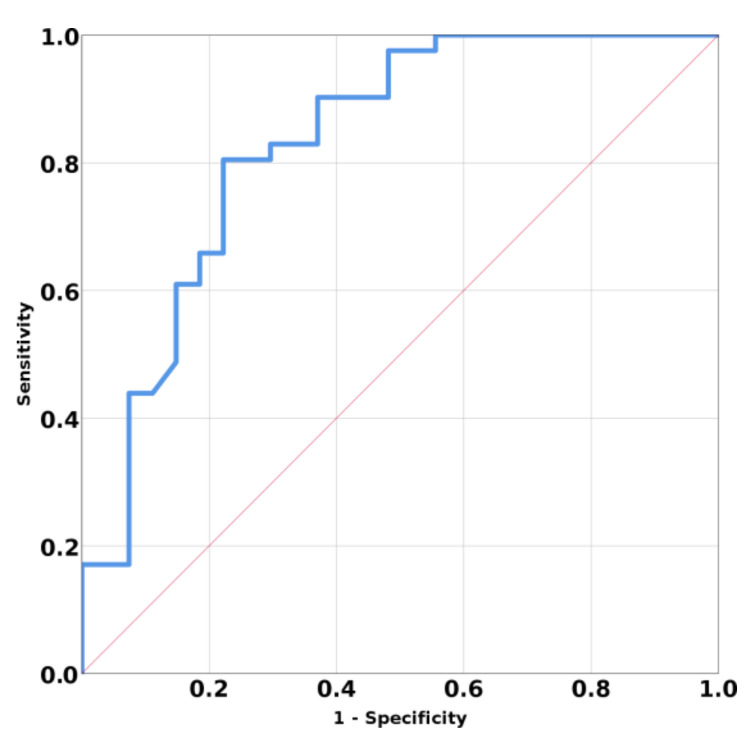
Area under curve (AUC) for prediction means SWE of primary hyperparathyroidism versus secondary hyperparathyroidism.

The ROC curve for discriminating primary vs. secondary hyperparathyroidism based on SWE mean values have AUC 0.831, 95% CI (0.725; 0.936); *p* < 0.001 (Figure 4). A mean SWE parathyroid value below 5.96 kPa has a specificity of 77.8%, a sensitivity of 80.5%, an accuracy of 77.9%, and a positive predicting value of 84.6% in identifying a parathyroid adenoma.

**Figure 5:**
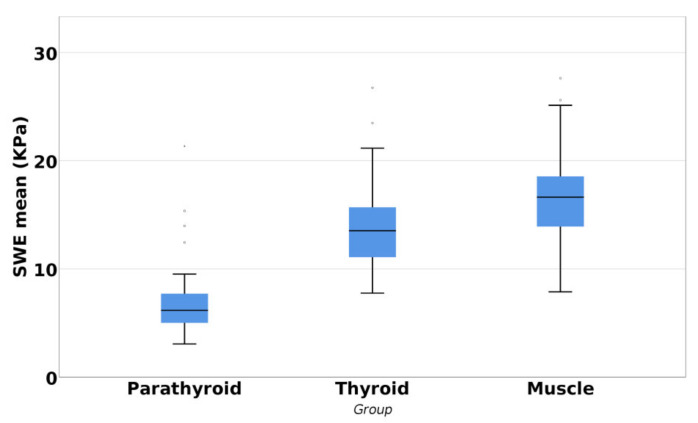
Differences between parathyroid, thyroid, and muscle tissue.

The Kruskal–Wallis test showed a statistically significant difference in the SWE mean values between parathyroid, thyroid, and muscle (*p* < 0.001). The parathyroid gland presented a higher elasticity when compared with the thyroid gland or muscle tissue (Figure 5).

**Figure 6:**
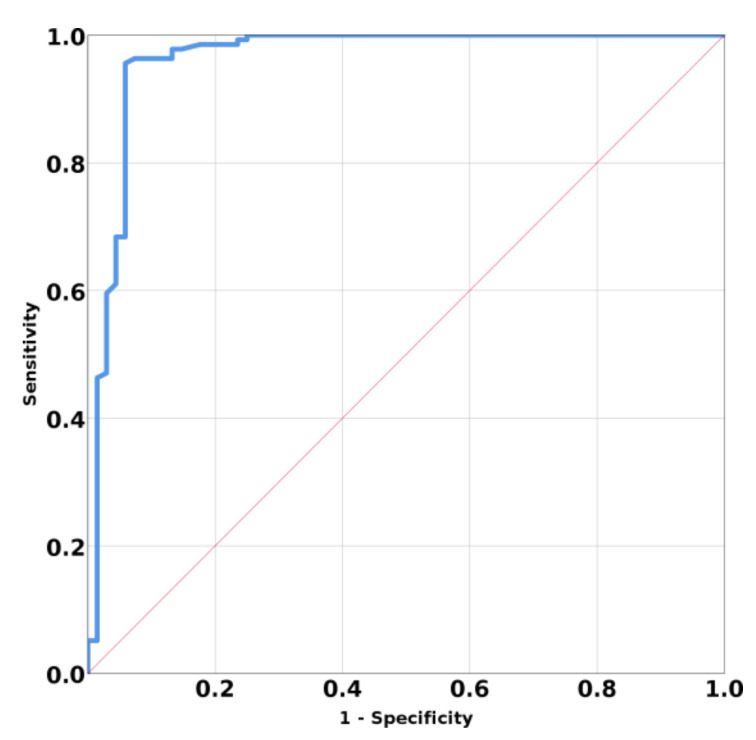
Mean SWE elastography values for parathyroid tissue compared with surrounding tissues.

The ROC curve for parathyroid tissue reported and thyroid and muscle tissue have AUC 0.953; 95% CI (0.928; 0.997); *p* < 0.001 (Figure 6). The determined cutoff value for parathyroid tissue was established below 9.58 kPa, with a sensitivity of 95.6%, a specificity of 94.1%, accuracy of 94.6%, negative predictive value of 94.4%, and positive predictive value of 97%.

## 4. Discussions

The endpoint of this study is to determine the elastographic characteristics of the parathyroid tissue in primary and secondary hyperparathyroidism and to evaluate the differences between them, even if adjusted to the nonparametric criterion. It also attempts to evaluate the differences between thyroid and muscle tissue for a better identification of the parathyroid tissue, adding value to the presurgical identification and diagnostic.

The role of the elastographic techniques in the parathyroid field has been evaluated, but it has not been yet established in the literature [40,41,42,43,44,45]. In the case of renal secondary hyperparathyroidism, there is not “when and how” applied for the parathyroid ultrasound and elastographic examination. Because of the frequency of this endocrine disease and the compulsory discriminative diagnostic, identification and localization before definitive treatment are mandatory.

The results were structured into two parts; in the first part of the results section, we evaluated the elastographic results by dividing them into two subgroups—the first group of primary hyperparathyroidism and the second group of secondary hyperparathyroidism—and we compared them, also including thyroid and muscle evaluation. In the second part of the results section, we evaluated the entire study group in order to assess the differences between the parathyroid, thyroid, and muscle tissue to obtain a final cutoff value for each studied tissue. The first group of parathyroid adenomas included 25 women and 2 men, as primary hyperparathyroidism is more prevalent in postmenopausal women [3,15].

As we have previously stated in our former studies on hyperparathyroidism (Shear Wave Elastography in Diagnosing Secondary Hyperparathyroidism, Diagnostics, 2019; Shear Wave Elastography versus Strain Elastography in Diagnosing Parathyroid Adenomas, Hindawi International Journal of Endocrinology Volume 2020), the mean SWE value is the best parameter to provide quantitative information on parathyroid adenomas. The significant elasticity differences between the parathyroid, thyroid, and muscle were confirmed in this paper, both for parathyroid adenomas and for parathyroid hyperplasia, the parathyroid tissue being significantly lower than the other two tissues.

As the pathophysiological mechanism is different in parathyroid adenoma from parathyroid hyperplasia from that of secondary renal hyperparathyroidism, we evaluated the differences amid the two subgroups, highlighting significant statistical differences among mean SWE and minimum SWE parathyroid value between parathyroid adenomas and parathyroid hyperplasia (*p* < 0.001) as well as for SWR parathyroid/thyroid and SWR parathyroid/muscle (*p* < 0.001).

By comparing the two types of hyperparathyroidism using the ROC curve, the best cutoff value for parathyroid adenoma is below 5.96 kPa, thus, emphasizing the fact that there are differences between primary and secondary hyperparathyroidism, when in doubt.

Keeping in mind the significant differences, even when we adjusted our PTH values for age, the difference of PTH serum levels remains significant, resulting that the age has no influence over PTH values.

In the literature, there are multiple research studies on the elastographic value of primary hyperparathyroidism, but this method is approached in fewer studies on secondary hyperparathyroidism.

Literature studies in the field of primary hyperparathyroidism have established different threshold values for parathyroid adenomas, depending on the elastography techniques used. Using shear wave virtual touch imaging quantification, with higher levels for parathyroid adenomas (2.16 ± 0.33 m/s) than parathyroid hyperplasia (1.75 ± 0.28 m/s), establishing a cut-off value greater than 1.92 m/s for parathyroid adenomas [41], by using the same elastography technique, another study compared parathyroid adenomas with thyroid tissue finding that parathyroid adenoma has a lower shear wave velocity value than thyroid tissue (2.01 m/s, respectively 2.77 m/s) [40].

Using ARFI imaging 2D SWE, a literature study compared parathyroid adenomas with malignant and benign thyroid pathology, determining that parathyroid adenomas with a mean SWV value of 3.09 ± 0.75 m/s, present a higher elasticity index than benign thyroid pathology, presented with a mean SWV of 2.20 ± 0.39 m/s and a higher elasticity than malignant thyroid lesions with a mean SWV of 3.59 ± 0.43 m/s [42].

Using 2D SWE elastography, with similar results to our results, a study conducted on parathyroid adenomas and benign thyroid nodules has concluded that parathyroid adenomas, with a mean SWE of 5.2 ± 7.2 kPa, presented a significantly lower elasticity index than benign thyroid nodules with a mean SWE of 24.3 ± 33.8 kPa [44]. 

Several elastographic studies on parathyroid hyperplasia have been published, but not focused on patients on renal replacement therapy. There are no threshold values for secondary hyperparathyroidism, in terms of parathyroid gland dimensions and volume, with a mention that a parathyroid diameter of more than 10 mm is suggestive for medical treatment [46].

To our knowledge, this is the first study performed with shear wave elastography that compared elastographic results of primary hyperparathyroidism and secondary hyperparathyroidism and compared the elastographic result with healthy thyroid and muscle tissue.

There are certain limitations to our study, first of all, the small number of patients enrolled in our study. However, we have to take into consideration for the primary hyperparathyroidism group that there is a time limit between diagnostic and surgical treatment, thus we cannot have a big delay between the diagnosis and treatment. For the secondary hyperparathyroidism group, we have evaluated an entire cohort of patients from a local dialysis center; however, we do not have pathology reports in all patients, as some present contraindications or did not accept parathyroid surgery. Taking into account the epidemiological situation we are confronting at the moment, further patient evaluations are difficult.

The clinical implications regarding the role of elastography in evaluating hyperparathyroidism, whether it is primary or secondary hyperparathyroidism is unequivocal. Complementary to conventional ultrasonography, elastography is a simple, non-invasive, repeatable, and reproducible method that can improve diagnosis and preoperative evaluation of the patient with either primary or secondary hyperparathyroidism.

As there are significant differences between primary and secondary hyperparathyroidism, we can establish a mean SWE cut-off value below 5.96 kPa to be specific for primary hyperparathyroidism.

If considering both parathyroid adenomas and parathyroid hyperplasia, we can establish a mean SWE cut-off value for parathyroid tissue below 9.58 kPa, with a significant difference between thyroid tissue, and muscle tissue.

Other studies in the field could help establish the elastographic differences between pathological parathyroid tissue and thyroid nodules. We do have to keep in mind, that literature studies have concluded that the elastography index of benign and malignant thyroid nodules is higher than the elasticity index of healthy thyroid tissue, in this way we can assume that if the parathyroid elastographic index is lower than healthy thyroid index, then it should be lower than benign and malignant thyroid nodules elasticity index.

## 5. Conclusions

In conclusion, the aim of this study was to identify and quantify the value of two-dimensional shear wave elastography in identifying, localizing and diagnosing hyperparathyroidism and to identify the elastographic differences between primary and secondary hyperparathyroidism. Elastography has been proven and validated in many clinical areas, including thyroid disease and it definitely presents an important role in the localization of the parathyroid disease. It can be a useful tool, qualitative, but mainly quantitative in offering a better differentiation of parathyroid tissue. We have to keep in mind that there are significant elastographic differences between parathyroid adenoma and parathyroid hyperplasia, but in either case, the parathyroid tissue is significantly lower than the healthy thyroid tissue and the surrounding muscle tissue.

## Figures and Tables

**Table 1:** Baseline characteristics of the studied subgroups.

Characteristics	Primary Hyperparathyroidism	Secondary Hyperparathyroidism	*p*-Value
Male to female ratio	2/25	22/19	<0.001
Age (years)	61 (48.67)	58 (50–65.5)	0.763
Parathormone (PTH) (pg/mL)	160.6 (115.0–206.3)	1117 (785.95–1407)	<0.001
Serum Phosphorus (mg/dL)	2.60 (2.30–3.12)	6.00 (5.15–7.77)	<0.001
Total Serum Calcium (mg/dL)	10.50 (10.10–11.40)	9.00 (8.35 –9.35)	<0.001
Serum vitamin D (ng/mL)	21.33 (15.55–25.52)	35.80 (24.20–44.70)	<0.001
Parathyroid volume (mL)	0.120 (0.068–0.240)	0.251 (0.109–0.332)	0.107
Maximum diameter (mm)	8.30 (6.20–12.00)	9.50 (7.25–11.75)	0.543
Dialysis years	-	5.10 (4.00–8.10)	NA
Kt/v	-	1.350 (1.30–1.46)	NA

**Table 2:** Baseline characteristics of the entire studied group. SWE: shear wave elastography.

Characteristics	Entire Study Group
Age	58 (50–66.50)
Serum PTH pg/mL	762.80 (190.30–1243.00)
Serum Phosphorus mg/dL	4.950 (2.725–6.625)
Total Serum Calcium mg/dL	9.40 (8.825–10.20)
Serum Vitamin D ng/mL	24.40 (20.35–38.52)
Parathyroid volume mL	0.180 (0.0852–0.311)
Mean SWE parathyroid	6.17 (4.995–7.745)
Min SWE parathyroid	3.38 (0.865–5.29)
Max SWE parathyroid	10.90 (9.075–13.56)
Mean SWE thyroid	13.53 (11.04–15.69)
Min SWE thyroid	8.55 (6.78–11.50)
Max SWE thyroid	18.77 (15.855–22.65)
Mean SWE muscle	16.63 (13.875–18.625)
Min SWE muscle	11.58 (8.815–14.075)
Max SWE muscle	22.61 (18.36–14.075)
SWE ratio parathyroid/thyroid tissue	0.4530 (0.3625–0.5975)
SWE ratio parathyroid/muscle tissue	0.3700 (0.2875–0.5275)

**Table 3:** Results of the first lot of primary hyperparathyroidism.

Evaluated Tissue	Mean SWE (kPa)	Min SWE (kPa)	Max SWE (kPa)
Parathyroid tissue	4.86 (3.42–5.84)	0.540 (0.140–1.36)	10.46 (9.44–12.76)
Thyroid tissue	12.08 (10.48–14.78)	6.96 (5.78–10.14)	18.04 (14.36–21.10)
Muscle tissue	16.86 (15.08–17.74)	11.80 (7.58–13.54)	27.50 (20.62–30.30)

**Table 4:** Results of the second lot of secondary hyperparathyroidism.

Evaluated Tissue	Mean SWE (kPa)	Min SWE (kPa)	Max SWE (kPa)
Parathyroid tissue	6.96 (6.09–8.44)	3.94 (3.38–5.75)	11.18 (8.98–13.68)
Thyroid tissue	14.22 (11.50–16.39)	9.38 (8.03–11.82)	20.00 (17.05–23.39)
Muscle tissue	15.66 (12.68–18.95)	11.44 (10.21–14.58)	21.22 (17.15–27.75)

**Table 5:** Elastography results in primary vs. secondary hyperparathyroidism. SWE: shear wave elastography.

Elastographic Measuments	Primary Hyperparathyroidism	Secondary Hyperparathyroidism	*p*-Value
Mean SWE parathyroid	4.86 (3.42–5.84)	6.96 (6.09–8.44)	<0.001
Min SWE parathyroid	0.540 (0.140–1.36)	3.94 (3.38–5.75)	<0.001
Max SWE parathyroid	10.46 (9.44–12.76)	11.18 (8.98–13.68)	0.590
Mean SWE thyroid	12.08 (10.48–14.78)	14.22 (11.50–16.39)	0.240
Min SWE thyroid	6.96 (5.78–10.14)	9.38 (8.03–11.82)	0.002
Max SWE thyroid	18.04 (14.36–21.10)	20.00 (17.05–23.39)	0.093
Mean SWE muscle	16.86 (15.08–17.74)	15.66 (12.68–18.95)	0.491
Min SWE muscle	11.80 (7.58–13.54)	11.44 (10.21–14.58)	0.940
Max SWE muscle	27.50 (20.62–30.30)	21.22 (17.15–27.75)	0.640
SWE ratio parathyroid/thyroid tissue	0.4040 (0.324–0.486)	0.520 (0.420–0.660)	<0.001
SWE ratio parathyroid/muscle tissue	0.2960 (0.226–0.372)	0.452 (0.348–0.667)	<0.001
